# Supplementary material for: Status, reporting completeness and methodological quality of pilot randomised controlled trials in acupuncture: protocol for a systematic review
Source: BMJ Open. 2021 Dec 3;11(12):e052528. doi: 10.1136/bmjopen-2021-052528 (PMC8647552; doi:10.1136/bmjopen-2021-052528)
Supplement: Author's manuscript [file bmjopen-2021-052528.draft_revisions.pdf]

BMJ Open is committed to open peer review. As part of this commitment we make the peer review history of every article we publish publicly available.

When an article is published we post the peer reviewers' comments and the authors' responses online. We also post the versions of the paper that were used during peer review. These are the versions that the peer review comments apply to.

The versions of the paper that follow are the versions that were submitted during the peer review process. They are not the versions of record or the final published versions. They should not be cited or distributed as the published version of this manuscript.

BMJ Open is an open access journal and the full, final, typeset and author-corrected version of record of the manuscript is available on our site with no access controls, subscription charges or pay-per-view fees (http://bmjopen.bmj.com).

If you have any questions on BMJ Open's open peer review process please email info.bmjopen@bmj.com

BMJ Open

Status and reporting quality of acupuncture pilot trials: a study protocol

Journal:	*BMJ Open*
Manuscript ID	bmjopen-2021-052528
Article Type:	Protocol
Date Submitted by the Author:	18-Apr-2021

Complete List of Authors:	Zhang, Yajun; Zhejiang Chinese Medical University,<br>Hu, Hantong; acupuncture department, The Third Affiliated Hospital of Zhejiang Chinese Medical University,; acupuncture department, The Third Affiliated Hospital of Zhejiang Chinese Medical University,<br>Li, Xiaoyu; Zhejiang Chinese Medical University<br>Lou, Jiali; Zhejiang Chinese Medical University<br>He, Xiaofen; Zhejiang Chinese Medical University<br>Jiang, Yongliang; Zhejiang Chinese Medical University, Key Laboratory of Acupuncture and Neurology of Zhejiang Province, Department of Neurobiology and Acupuncture Research, The Third Clinical Medical College<br>Fang, Jian-Qiao; Key Laboratory of Acupuncture and Neurology of Zhejiang Province, Department of Neurobiology and Acupuncture Research

Keywords:	COMPLEMENTARY MEDICINE, Protocols & guidelines < HEALTH SERVICES ADMINISTRATION & MANAGEMENT, STATISTICS & RESEARCH METHODS, Clinical trials < THERAPEUTICS

SCHOLARONE™
Manuscripts

BMJ

I, the Submitting Author has the right to grant and does grant on behalf of all authors of the Work (as defined in the below author licence), an exclusive licence and/or a non-exclusive licence for contributions from authors who are: i) UK Crown employees; ii) where BMJ has agreed a CC-BY licence shall apply, and/or iii) in accordance with the terms applicable for US Federal Government officers or employees acting as part of their official duties; on a worldwide, perpetual, irrevocable, royalty-free basis to BMJ Publishing Group Ltd ("BMJ") its licensees and where the relevant Journal is co-owned by BMJ to the co-owners of the Journal, to publish the Work in this journal and any other BMJ products and to exploit all rights, as set out in our licence.

The Submitting Author accepts and understands that any supply made under these terms is made by BMJ to the Submitting Author unless you are acting as an employee on behalf of your employer or a postgraduate student of an affiliated institution which is paying any applicable article publishing charge ("APC") for Open Access articles. Where the Submitting Author wishes to make the Work available on an Open Access basis (and intends to pay the relevant APC), the terms of reuse of such Open Access shall be governed by a Creative Commons licence – details of these licences and which Creative Commons licence will apply to this Work are set out in our licence referred to above.

Other than as permitted in any relevant BMJ Author's Self Archiving Policies, I confirm this Work has not been accepted for publication elsewhere, is not being considered for publication elsewhere and does not duplicate material already published. I confirm all authors consent to publication of this Work and authorise the granting of this licence.

For peer review only

Status and reporting quality of acupuncture pilot trials: a study protocol

Yajun Zhang[a], Hantong Hu[ab], Xiaoyu Li[a], Jiali Lou[a], Xiaofen He[a], Yongliang Jiang[a], Jianqiao Fang[a,*]

[a]Department of Neurobiology and Acupuncture Research, The Third Clinical Medical College, Zhejiang Chinese Medical University, Key Laboratory of Acupuncture and Neurology of Zhejiang Province, Hangzhou 310053, China

[b]Department of acupuncture and moxibustion, The Third Affiliated Hospital of Zhejiang Chinese Medical University, Hangzhou City, Zhejiang Province, China

***Correspondence**

Prof. Jianqiao Fang, Department of Neurobiology and Acupuncture Research, The Third Clinical Medical College, Zhejiang Chinese Medical University, Key Laboratory of Acupuncture and Neurology of Zhejiang Province, Hangzhou 310053, China; Email: fangjianqiao7532@163.com; Fax number: 86-571-88393504

For peer review only

Abstract

Introduction: There have been no published studies that aimed to investigate the status of pilot trials in the acupuncture field. Key questions concerning the publication trends, impacts, feasibility, reporting quality of acupuncture pilot trials are urgently to be solved. Thus, this study presents a protocol that aims to: (1) investigate publication trends as well as aspects of feasibility evaluated in acupuncture pilot trials; (2) identify the proportion of pilot trials that lead to definitive trials; and (3) assess the quality of reporting in pilot trials in the acupuncture field.

Methods and analysis: Studies of acupuncture pilot trials indexed in 7 databases published from 2011 to 2021 will be included. Two independent investigators will conduct study selection, classify the aspects of feasibility, and assess the reporting quality of each included study based on the CONSORT extension to randomized pilot and feasibility trials. For the primary analysis, aspects of feasibility being assessed in the included pilot trials will be tabulated. The proportion of pilot trials that lead to definitive trials will also be calculated. Moreover, a quantitative analysis of the quality in reporting of acupuncture pilot trials will be conducted. The secondary analysis will adopt a regression analysis to identify factors associated with the reporting quality.

Ethics and dissemination: Ethical approval is not required for this study. This study is planned to be submitted to an academic journal for peer reviews.

Review registration: Open Science Framework https://osf.io/9kmb3/

For peer review only

Strengths and limitations of this study

1. This protocol will be the first study that aimed to investigate the status as well as the methodological quality of pilot trials in the acupuncture field based on the CONSORT extension for reporting randomized pilot and feasibility trials.
2. The investigation in the completeness and transparency in reporting acupuncture pilot studies based on the CONSORT checklist will lead to a better understanding of the methodological quality of current studies, by which it will shed the light to promote the design and reporting quality of acupuncture pilot trials in the future.
3. The main limitation is that there may be missing or unavailable data to determine why acupuncture pilot trials have not led to subsequent definitive trials.

1. Introduction

Although clinical trials serve as the basis for improvement of clinical practice and establishment of evidence-based clinical guidelines, they generally consume considerable human, financial, and material resources. Moreover, it is difficult to make major changes once they are conducted formally[1]. In such situations, if the design of the formal trial has major flaws, it will lead to wasting of resources. A previously published study[2] estimated that 85% of research funds for biomedical research are wasted each year due to misdirected or poorly designed studies. Therefore, it is of significance to validate and improve the scientific questions, trial designs and protocol implementation by conducting pilot trials prior to a formal trial[3].

Despite a number of definitions of pilot trials have been put forward, most of them have in common the idea of conducting a study prior to a larger, more comprehensive study[4]. For example, the UK National Institute for Health Research (NIHR) defines a pilot trial explicitly as ‘a version of the main study that is run in miniature to test whether the components of the main study can all work together, which is a widely accepted definition[4]. Therefore, whether a study can be labelled as a pilot trial mainly depends on the primary objective of the study, which is to evaluate

For peer review only

the feasibility and coordination of the components of an ensuing definitive trial.

Given that the design of a pilot trial is generally consistent with the subsequent formal trial, a well-designed pilot trial can not only identify potential problems arisen in a formal trial in advance, but also promote the formal trial by investigating the feasibility and coordination of the study design, thus avoiding potentially disastrous consequences if large-scale trials are conducted rashly[5]. In the guidelines of the UK Medical Research Council (MRC) for designing and evaluating complex interventions, pilot studies are explicitly recommended, particularly in relation to identifying problems that might arise in a subsequent large-scale trial of a complex intervention[6, 7].

Acupuncture belongs to complex interventions, so it is more necessary to conduct pilot trials before a formal acupuncture trial. In recent years, the benefits of pilot trials have been verified in a number of high-quality clinical studies of acupuncture. For example, based on results obtained from a series of pilot trials concerning acupuncture for treating osteoarthritis[8-10], Liu et al. not only fully examined the study procedure to determine the feasibility of study designs, but also obtained important pilot data, such as optimal acupuncture frequency, patient compliance rate, and primary outcome measures. Subsequently their research team improved the design of the large-scale definitive trial and implemented it successfully, which was published in Arthritis & Rheumatology[11].

Despite notable value and impacts, the role of pilot studies remains underestimated in scientific research. Moreover, previous studies demonstrated that only a very small percentage of pilot trials lead to definitive trials[12]. To our knowledge, there have been no published studies that aimed to investigate the status and methodological quality of pilot trials in the acupuncture field. Key questions concerning the publication trend, impact, feasibility, and reporting quality of acupuncture pilot trials are urgently to be solved. Thus, this study protocol is designed and aims to: 1) investigate the overall publication trends as well as the aspects of feasibility evaluated in acupuncture pilot trials; 2) identify the proportion of pilot trials that lead to definitive trials; 3) assess the quality of reporting in pilot trials in the

For peer review only

acupuncture field.

2. Methods

2.1 Eligibility criteria

A previously published tutorial[5] regards that 'pilot' trials belong to the dimension of 'feasibility' trials and a pilot trial is synonymous with a feasibility study that is intended to guide the planning of a large-scale investigation. Based on the main goals of this review and references of similar studies[13-15], the boundary between pilot and feasibility trials will not be imposed in our study. We use the term 'pilot' trial to refer to the studies that will be reviewed here and the term 'feasibility' if the original authors of the included publications adopted this term rather than 'pilot'. Therefore, published trials will be eligible if studies were defined and reported explicitly as pilot trials within the published paper itself by the original authors. Additionally, in reference to the similar studies[13-15], publications labelled as 'feasibility', 'preliminary', 'exploratory', 'proof-of-concept' and 'vanguard' studies/trials' will also be considered for eligibility because these terms are often used interchangeably with the term 'pilot'[12, 16, 17]. In addition, given that the International Committee of Medical Journal Editors proposed that all trials conducted after July 2005 should be registered in a public registry platform[18, 19] and the latest update of the Consolidated Standards of Reporting Trials (CONSORT) statement was released in 2010[20], only publications of pilot trials published after 2010 will be evaluated for eligibility in our review.

Studies reported as acupuncture pilot or feasibility trials will be eligible for this review if they meet the following criteria: 1) included the evaluation of at least one kind of acupuncture interventions; 2) randomized control trials (RCTs) conducted in humans; 3) trials published from 2011 to 2021 in any language. The term 'acupuncture' in this review is defined as interventions involving penetration of specific points on the skin by needling, regardless of needling and stimulation modalities, such as manual acupuncture, electroacupuncture, auricular acupuncture and warm needling, or a combination of these modalities. Studies will be excluded if:

For peer review only

1) they are non-RCTs or there is no control groups, such as trials with a pre–post design or an observational study design; 2) no acupuncture intervention was used solely or in combination in the trial.

2.2 Databases and search strategy

To search published studies of acupuncture pilot trials, we will conduct a comprehensive retrieval on 4 English databases (PubMed, Web of Science, EMBASE, Cochrane Library) and 3 Chinese databases (Chinese National Knowledge Infrastructure, Wanfang Database, Chinese Biomedical Literature Database). The time range of the published pilot trials will be limited within 2011 and June 2021.

Search strategies will consist of two components: interventions (manual acupuncture, electroacupuncture, etc) and study type (pilot trial). Search strategies will combine subject headings (e.g. Medical Subject Headings [MeSH] for PubMed) and keywords (acupuncture, eletroacupuncture, pilot, feasibility, preliminary, exploratory, proof-of-concept, vanguard, etc). The PubMed retrieval strategy will be adapted for other databases by replacing MeSH terms with proper subject headings (if applicable) and maintaining the same keywords. The retrieval strategy for PubMed is presented in Table 1. All retrieval results will be imported into the Mendeley Reference Manager software (Elsevier Publishing, 2013).

With all the included pilot trials determined, a secondary search will be performed in the aforementioned databases to identify corresponding future definitive trials. The secondary search will be conducted using the keywords in the pilot trials. In addition, the International Clinical Trials Registry Platform (ICTRP), Clinicaltrials Registry (https://clinicaltrials.gov/), and Chinese Clinical Trial Registry (http://www.chictr.org.cn) will be searched to ascertain whether a future definitive trial was conducted (or in progress) following the previously included pilot trial. Finally, if no future definitive trials are identified based on these retrieval methods, we will try to contact the corresponding authors of the published pilot study by email and query whether a subsequent full-scale definitive trial was in progress, conducted, or submitted for publication.

For peer review only

2.3 Screening and study selection

Following the removal of duplicate retrieval literature, two investigators will scan titles and abstracts of retrieval studies independently to determine studies that claim to be acupuncture pilot or feasibility trials. In the first round screening, two independent investigators will browse the titles and abstracts of self-claimed acupuncture pilot or feasibility trials to confirm eligibility. In the second roung screening, the entire text of potentially qualified studies will be downloaded to determine eligibility based on full-text reading. A third investigator will be invited to settle any disagreements between the two investigators arisen in the course of study extraction. The flowchart of study search and screening is presented in Figure 1

2.4 Data extractions

A pre-designed electronic data extraction form is developed for this study based on the recommendation from the CONSORT group[20]. Two investigators will extract data or information of all included trials independently using the data extraction form, which consists of various aspects as follows: 1) the language of publication; 2) the country that conducts the trial; 3) publication year; 4) study designs; 5) conditions being treating; 6) the types of intervention; 6) the types of controls ; 7) trial registration platforms (if applicable); 8) the impact factor of the journal (if applicable) in the publication year based on the Journal Citation Reports (https://jcr.incites.thomsonreuters. com); 9) the number of authors; 10) sample sizes; 11) the source of funding; and so on.

With the attempt to improve consistency and accuracy between the investigators, prior to full-scale data extraction, the feasibility of data extraction forms and between-reviewer agreements will be investigated via kappa coefficients by choosing 10 randomly selected studies. If inadequate consistency and accuracy are identified, extra training on data extraction will be conducted. A third reviewer will be invited to verify the accuracy of all extracted data and settle any discrepancies between the two investigators arisen in the course of data extraction.

For peer review only

2.5 Assessment of the aspects of feasibility

(1) The rationales for conducting a pilot trial.

We will classify whether the original authors of the pilot study stated the rationales for conducting a pilot trial (1 for yes or 0 for no). If yes, we will further classify the rationales for conducting pilot and feasibility studies based on a widely acknowledged tutorial, which suggests 4 broad classifications in general to sort out the rationales for performing pilot trials as follows[5].

1) Process: this invlolves the assessment of the feasibility of the steps that need to take place as part of the future definitive trial, such as estimation of recruitment rates, retention rates, etc.

2) Resources: concerning the evaluation of budget and time problems that might arise during the future definitive trial.

3) Management: this involves the evaluation of potentially human and data optimization problems, such as personnel and data management issues in different research centres.

4) Scientific: this involves the evaluation of treatment safety, determination of treatment dose, and estimation of treatment effect size, such as acquisition of preliminary data for sample size estimation in the future definitive trial

If there is more than one classification identified for an included acupuncture pilot trial, all applicable classifications will be coded.

(2) the conclusion of a pilot trial

In general, the conclusions of a pilot or feasibility trial can be grouped under several classifications as follows[15]: 1) the study protocol is not feasible and the future full-scale definitive trial is not recommended to be conducted; 2) the study protocol is feasible to a certain extent but needs to be modified before proceeding to a future full-scale definitive trial; 3) the study protocol does not need to be modified, but the future full-scale definitive trial needs to be performed under close monitoring; 4) the study protocol is completely feasible.

We will classify the conclusion of each included pilot trial according to the above-mentioned classifications.

For peer review only

(3) Whether a future definitive trial was conducted

Through a secondary search in the aforementioned databases and trials registry platforms or an attempt to contact the original authors of the pilot trial by email, we will classify whether a a definitive trial was conducted following the pilot trial (1 for yes or 0 for no). Meanwhile, we will investigate whether the pilot data were used for sample size calculation in the follow-up definitive trial (1 for yes or 0 for no).

2.6 Assessment of reporting quality in pilot trials

Two independent investigators will evaluate the reporting quality of each included studies in accordance with the CONSORT extension for reporting randomized pilot and feasibility trials, which consist of a 40-item checklist[21]. The 40-item checklist of CONSORT mainly consists of domains in terms of the title, abstract, objectives, trial design, methods, results, conclusions, limitations, registrations and funding[22]. In our study, every item of the CONSORT checklist will be classified as 'reported', 'inadequately reported', 'not reported' or 'not applicable'. Total score of the CONSORT checklist (range 0–40) extension for reporting randomized pilot and feasibility trials of each included study will be calculated based on the reporting of the published studies.

2.7 Statistical analysis

For the primary analysis, the number of publications per year will be calculated and presented in a tendency chart to analyze overall publication trends of acupuncture pilot trials. The aspects of feasibility assessed in the included pilot trials will be tabulated. The proportion of pilot trials that lead to follow-up definitive trials will be calculated. A quantitative analysis of the quality in reporting of acupuncture pilot trials will be conducted. In details, the frequency regarding each item of the CONSORT checklist rated as reported, inadequately reported, not reported and not applicable will be computed. The average total score for the CONSORT checklist will be calculated and displayed as mean ± standard deviation (SD).

For the secondary analysis, regression analysis will be applied to determine

which study characteristic (e.g. language of publication, the number of authors, trial registration or not, trial funded or not) has associations with the reporting quality of acupuncture pilot trials. A p-value less than 0.05 is considered significant. All analyses will be performed using SPSS 25.0 software (IBM, USA).

2.8 Patient and public involvement

Patients and the public will not involve in this study.

3. Discussion

Based on a brief review of acupuncture studies published in journals with high impact factors in recent years, the majority of them are conducted based on well-designed pilot trials that were also published earlier. For example, Liu et al. conducted an acupuncture pilot trial[23] and found that electroacupuncture at Zhongliao (BL33) and Huiyang (BL35) could lead to less urine leakage in women with stress urinary incontinence with an acceptable safety and feasibility profile. This pilot study[23] provided evidence for the acupoint selection and feasibility for a subsequent multicenter RCT of electroacupuncture for treating female stress incontinence published in JAMA[24]. Additionally, Liu et al. also performed a series of pilot trials investigating the effect of acupuncture on functional constipation[25-27], which provided a solid basis for the subsequent definitive trial of acupuncture for chronic severe functional constipation, which was published in Annals of Internal Medicine[28]. Such phenomena indicate the acupuncture researchers are paying increasing attention to the value of pilot trials.

Neverthess, to the best of our knowledge, there have been no previously published studies conducted to investigate the status and methodological quality of pilot trials in the acupuncture field, this protocol will be the first study that aimed to investigate the status as well as the methodological quality of pilot trials in the acupuncture field. Given that the pilot trial is a key component of clinical research to obtain important preliminary data and information for improving the study design and methodological quality of future definitive trials, this study is of great importance in

For peer review only

the filed of acupuncture and will guide acupuncture clinical research for both researchers and clinicians in the future.

Acupuncture belongs to complex treatment interventions. Due to the characteristic of acupuncture treatment, patients with specific diseases may be unwilling or unable to receive acupuncture treatment, especially in western countries. Thus, before the formal trial, preliminary data and information are very valuable, such as the selection of appropriate acupuncture points, the treatment frequency, the recruitment rate and dropout rate are urgently to be obtained prior to a subsequent definitive trial. The interpretation of these preliminary results will directly determine whether a future full-scale trial should be conducted, or whether the current study protocol needs modification. It can also provide pilot data for sample size calculation in the future definitive trial.

The first strength of this review is to conduct a broad retrieval to identify all eligible acupuncture pilot trials published from 2011 to 2021, based on which the status and publication trends will be revealed. Secondly, this review will assess the reporting quality of each included pilot trial in accordance with the 40-item CONSORT extension for reporting randomized pilot and feasibility trials[21], which is a highly acknowledged and recommended tool[29]. Transparent and complete reporting is a key principle of rigorous research, and reporting tools (eg, CONSORT) enable both authors and readers to report and interpret studies. Specifically, the CONSORT extension for reporting randomized pilot and feasibility trials[21] is developed to provide reporting guidance for any randomized study in which a future definitive RCT, or part of it, is conducted on a smaller scale, regardless of its design (eg, cluster, crossover) or the terms used by authors to label the study (eg, pilot, feasibility). A previous systematic review indicated that the adoption of the CONSORT checklist has close associations with improvement in the quality of reporting of randomised controlled trials[30]. Thus, the investigation in the completeness and transparency in reporting acupuncture pilot studies based on the CONSORT checklist will lead to a better understanding of the methodological quality of current studies, by which it will shed the light to promote the design and reporting quality of acupuncture pilot trials in

For peer review only

the future.

The main limitation is that there may be missing or unavailable data to determine why acupuncture pilot trials have not led to subsequent definitive trials. In addition, given that CONSORT extension for reporting randomized pilot and feasibility trials is adopted to evaluate the reporting quality of included studies and the latest update of the CONSORT statement was released in 2010[20], only publications of pilot trials with a RCT design published after 2010 will be included for analysis in our review.

For peer review only

References

[1] Wang Y, Wang LQ, Yang JW, et al. Application and reflection on pilot study in acupuncture clinical research. Zhongguo Zhen Jiu. 2021;41(3):325-329.

[2] Chalmers I, Glasziou P. Avoidable waste in the production and reporting of research evidence. Lancet. 2009;374(9683):86-89.

[3] Moher D, Glasziou P, Chalmers I, et al. Increasing value and reducing waste in biomedical research: who's listening?. Lancet. 2016;387(10027):1573-1586.

[4] Eldridge SM, Lancaster GA, Campbell MJ, et al. Defining Feasibility and Pilot Studies in Preparation for Randomised Controlled Trials: Development of a Conceptual Framework. PLoS One. 2016;11(3):e0150205.

[5] Thabane L, Ma J, Chu R, et al. A tutorial on pilot studies: the what, why and how. BMC Med Res Methodol. 2010;10:1.

[6] Craig P, Dieppe P, Macintyre S, et al. Developing and evaluating complex interventions: the new Medical Research Council guidance. Bmj. 2008;337:a1655.

[7] Lancaster GA, Campbell MJ, Eldridge S, et al. Trials in primary care: statistical issues in the design, conduct and evaluation of complex interventions. Stat Methods Med Res. 2010;19(4):349-377.

[8] Lin LL, Li YT, Tu JF, et al. Effectiveness and feasibility of acupuncture for knee osteoarthritis: a pilot randomized controlled trial. Clin Rehabil. 2018;32(12):1666-1675.

[9] Lin LL, Tu JF, Wang LQ, et al. Acupuncture of different treatment frequencies in knee osteoarthritis: a pilot randomised controlled trial. Pain. 2020;161(11):2532-2538.

[10] Wang TQ, Li YT, Wang LQ, et al. Electroacupuncture versus manual acupuncture for knee osteoarthritis: a randomized controlled pilot trial. 2020;38(5):291-300.

[11] Tu JF, Yang JW, Shi GX, et al. Efficacy of Intensive Acupuncture Versus Sham Acupuncture in Knee Osteoarthritis: A Randomized Controlled Trial. 2021;73(3):448-458.

For peer review only

[12] Arain M, Campbell MJ, Cooper CL, et al. What is a pilot or feasibility study? A review of current practice and editorial policy. BMC Med Res Methodol. 2010;10:67.

[13] Desai B, Desai V, Shah S, et al. Pilot randomized controlled trials in the orthopaedic surgery literature: a systematic review. BMC Musculoskelet Disord. 2018;19(1):412.

[14] Scola LFC, Moseley AM, Thabane L, et al. A methodological survey on reporting of pilot and feasibility trials for physiotherapy interventions: a study protocol. BMJ Open. 2019;9(5):e020580.

[15] Kaur N, Figueiredo S, Bouchard V, et al. Where have all the pilot studies gone? A follow-up on 30 years of pilot studies in Clinical Rehabilitation. Clin Rehabil. 2017;31(9):1238-1248.

[16] Arnold DM, Burns KE, Adhikari NK, et al. The design and interpretation of pilot trials in clinical research in critical care. Crit Care Med. 2009;37(1 Suppl):S69-74.

[17] Leon AC, Davis LL, Kraemer HC. The role and interpretation of pilot studies in clinical research. J Psychiatr Res. 2011;45(5):626-629.

[18] De Angelis C, Drazen JM, Frizelle FA, et al. Clinical trial registration: a statement from the International Committee of Medical Journal Editors. N Engl J Med. 2004;351(12):1250-1251.

[19] Costa LO, Lin CW, Grossi DB, et al. Clinical trial registration in physical therapy journals: Recommendations from the International Society of Physiotherapy Journal Editors. Phys Ther. 2013;93(1):6-10.

[20] Schulz KF, Altman DG, Moher D. CONSORT 2010 statement: updated guidelines for reporting parallel group randomised trials. BMJ. 2010;340:c332.

[21] Eldridge SM, Chan CL, Campbell MJ, et al. CONSORT 2010 statement: extension to randomised pilot and feasibility trials. Pilot Feasibility Stud. 2016;2:64.

[22] Thabane L, Hopewell S, Lancaster GA, et al. Methods and processes for development of a CONSORT extension for reporting pilot randomized

For peer review only

controlled trials. Pilot Feasibility Stud. 2016;2:25.

[23] Xu HF, Du RS, Mo Q, et al. A phase 1 clinical study on the efficacy of electroacupuncture for female patients with mild to moderate stress incontinence. China Journal of Traditional Chinese Medicine and Pharmacy. 2014;29(12):3755-3758.

[24] Liu Z, Liu Y, Xu H, et al. Effect of Electroacupuncture on Urinary Leakage Among Women With Stress Urinary Incontinence: A Randomized Clinical Trial. JAMA. 2017;317(24):2493-2501.

[25] Xue QM, Li N, Liu ZS, et al. Efficacy of electroacupuncture in the treatment of functional constipation: A randomized controlled pilot trial. Chin J Integr Med. 2015;21(6):459-463.

[26] Qu JN, Zhang BY, Xu HF, et al. Effi cacy of electro-acupuncture for severe functional constipation: A randomized controlled pilot trial. China Journal of Traditional Chinese Medicine and Pharmacy. 2014;29(05):1424-1428.

[27] Qu JN, Zhang BY, Zhu WZ, et al. Comparison of efficacy on functional constipation treated with electroacupuncture of different acupoint prescriptions: a randomized controlled pilot trial. Chinese Acupuncture and Moxibustion. 2014;34(06):521-528.

[28] Liu Z, Yan S, Wu J, et al. Acupuncture for Chronic Severe Functional Constipation: A Randomized Trial. Ann Intern Med. 2016;165(11):761-769.

[29] Thoma A, Coroneos CJ, Eaves FF. You Can't See What You Can't See: Transparency in RCT Reporting, and the Role of the CONSORT Checklist. Aesthet Surg J. 2020.

[30] Plint AC, Moher D, Morrison A, et al. Does the CONSORT checklist improve the quality of reports of randomised controlled trials? A systematic review. Med J Aust. 2006;185(5):263-267.

For peer review only

Funding

The trial is financially supported by the National Key R&D Program of China (NO.2018YFC1704600).

Competing interests

The authors declare that they have no competing interests.

Data availability statement

All data are available upon reasonable request

For peer review only

Figure legends

Figure 1. The flow diagram of study search and screening

For peer review only

Table 1. Search strategy in PubMed.

No.	Search items
#1	Randomized controlled trial [pt]
#2	Controlled clinical trial [pt]
#3	Randomized OR Randomised [Title/Abstract]
#4	Clinical trials [MeSH]
#5	Randomly [Title/Abstract]
#6	Trial [Title/Abstract]
#7	#1 OR #2 OR #3 OR #4 OR #5 OR #6
#8	Humans [MeSH]
#9	#7 AND #8
#10	Pilot Projects OR Feasibility Studies OR Proof of Concept Study [MeSH]
#11	pilot OR feasibility OR preliminary OR exploratory OR proof-of-concept OR vanguard [Title/Abstract]
#12	#10 OR #11
#13	Acupuncture Therapy [MeSH]
#14	(acupuncture OR electroacupuncture OR electro-acupuncture OR manual acupuncture OR auricular acupuncture OR ear acupuncture OR warm needling OR warming-needle moxibustion) [Title/Abstract]
#15	#13 OR #14
#16	#9 AND #12 AND #15

For peer review only

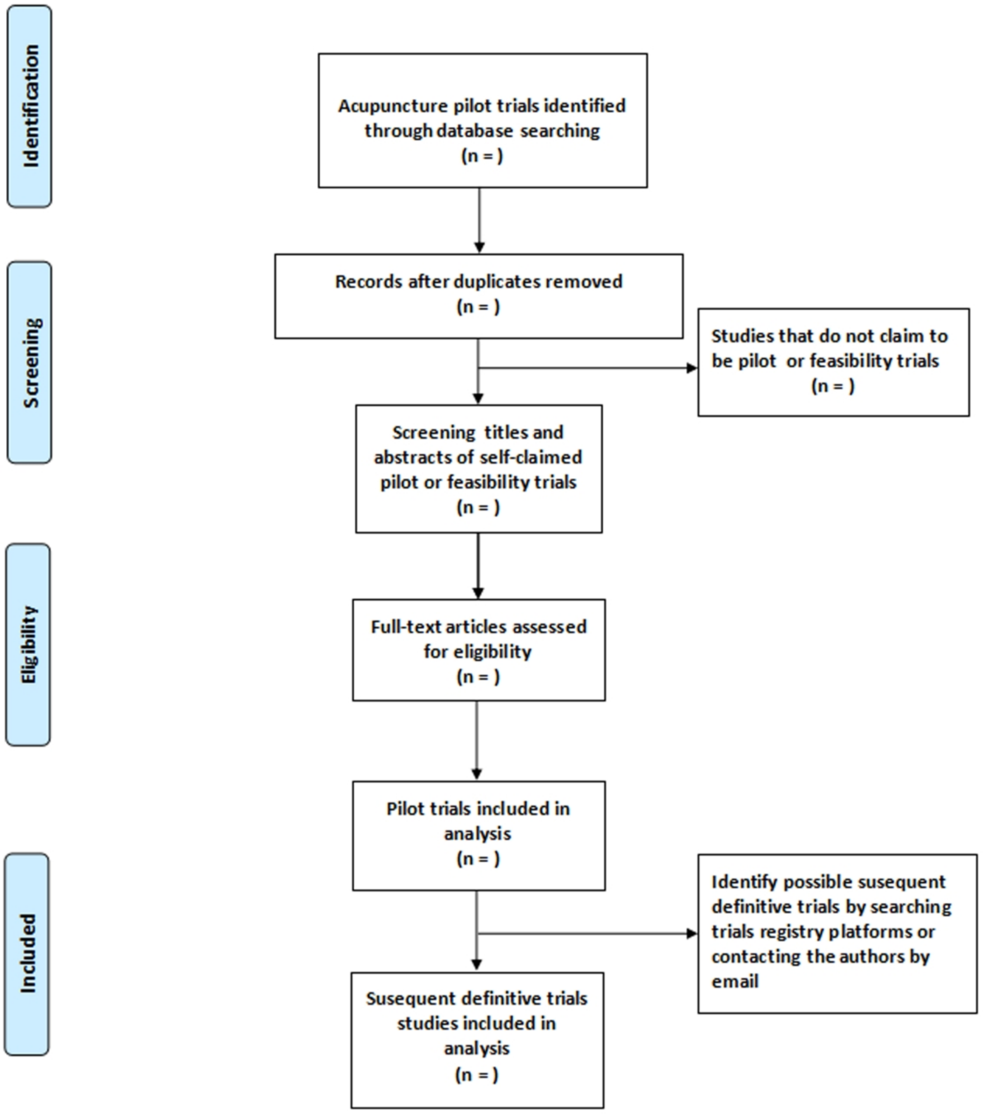

Figure 1. The flow diagram of study search and screening

226x256mm (300 x 300 DPI)

The status, reporting completeness and methodological quality of pilot randomized controlled trials in acupuncture: protocol for a systematic review

Journal:	*BMJ Open*
Manuscript ID	bmjopen-2021-052528.R1
Article Type:	Protocol
Date Submitted by the Author:	18-Oct-2021
Complete List of Authors:	Zhang, Yajun; Zhejiang Chinese Medical University,<br>Hu, Hantong; acupuncture department, The Third Affiliated Hospital of Zhejiang Chinese Medical University,; acupuncture department, The Third Affiliated Hospital of Zhejiang Chinese Medical University,<br>Li, Xiaoyu; Zhejiang Chinese Medical University<br>Lou, Jiali; Zhejiang Chinese Medical University<br>He, Xiaofen; Zhejiang Chinese Medical University<br>Jiang, Yongliang; Zhejiang Chinese Medical University, Key Laboratory of Acupuncture and Neurology of Zhejiang Province, Department of Neurobiology and Acupuncture Research, The Third Clinical Medical College<br>Fang, Jian-Qiao; Key Laboratory of Acupuncture and Neurology of Zhejiang Province, Department of Neurobiology and Acupuncture Research
**Primary Subject Heading**:	Complementary medicine
Secondary Subject Heading:	Qualitative research, Complementary medicine
Keywords:	COMPLEMENTARY MEDICINE, Protocols & guidelines < HEALTH SERVICES ADMINISTRATION & MANAGEMENT, STATISTICS & RESEARCH METHODS, Clinical trials < THERAPEUTICS

I, the Submitting Author has the right to grant and does grant on behalf of all authors of the Work (as defined in the below author licence), an exclusive licence and/or a non-exclusive licence for contributions from authors who are: i) UK Crown employees; ii) where BMJ has agreed a CC-BY licence shall apply, and/or iii) in accordance with the terms applicable for US Federal Government officers or employees acting as part of their official duties; on a worldwide, perpetual, irrevocable, royalty-free basis to BMJ Publishing Group Ltd ("BMJ") its licensees and where the relevant Journal is co-owned by BMJ to the co-owners of the Journal, to publish the Work in this journal and any other BMJ products and to exploit all rights, as set out in our licence.

The Submitting Author accepts and understands that any supply made under these terms is made by BMJ to the Submitting Author unless you are acting as an employee on behalf of your employer or a postgraduate student of an affiliated institution which is paying any applicable article publishing charge ("APC") for Open Access articles. Where the Submitting Author wishes to make the Work available on an Open Access basis (and intends to pay the relevant APC), the terms of reuse of such Open Access shall be governed by a Creative Commons licence – details of these licences and which Creative Commons licence will apply to this Work are set out in our licence referred to above.

Other than as permitted in any relevant BMJ Author's Self Archiving Policies, I confirm this Work has not been accepted for publication elsewhere, is not being considered for publication elsewhere and does not duplicate material already published. I confirm all authors consent to publication of this Work and authorise the granting of this licence.

For peer review only

The status, reporting completeness and methodological quality of pilot randomized controlled trials in acupuncture: protocol for a systematic review

Yajun Zhang[a1], Hantong Hu[ab1], Xiaoyu Li[a], Jiali Lou[a], Xiaofen He[a], Yongliang Jiang[a], Jianqiao Fang[a,*]

[a]Department of Neurobiology and Acupuncture Research, The Third Clinical Medical College, Zhejiang Chinese Medical University, Key Laboratory of Acupuncture and Neurology of Zhejiang Province, Hangzhou 310053, China

[b]Department of Acupuncture and Moxibustion, The Third Affiliated Hospital of Zhejiang Chinese Medical University, Hangzhou City, Zhejiang Province, China

[1] These authors contribute equally as co-first authors

***Correspondence**

Prof. Jianqiao Fang, Department of Neurobiology and Acupuncture Research, The Third Clinical Medical College, Zhejiang Chinese Medical University, Key Laboratory of Acupuncture and Neurology of Zhejiang Province, Hangzhou 310053, China; Email: fangjianqiao7532@163.com; Fax number: 86-571-88393504

For peer review only

Abstract

Introduction: To date, there has been a lack of knowledge about the status, reporting completeness, and methodological quality of pilot trials in the acupuncture field. Thus, this systematic review protocol aims to: (1) investigate publication trends and aspects of feasibility evaluated in acupuncture pilot trials; (2) identify the proportion of acupuncture pilot trials that lead to definitive trials; and (3) assess the reporting completeness and methodological quality of pilot trials in acupuncture.

Methods and analysis: Studies of acupuncture pilot randomized controlled trials published from 2011 to 2021 will be retrieved in 7 databases in January 2022, including PubMed, Web of Science, EMBASE, Cochrane Library, Chinese National Knowledge Infrastructure, Wanfang Database, and Chinese Biomedical Literature Database. The methodological quality and reporting completeness of all included studies will be assessed using the risk of bias 2.0 tool (RoB 2) and the Consolidated Standards of Reporting Trials (CONSORT) extension to randomized pilot and feasibility trials, respectively. For the primary analysis, publication trends, aspects of feasibility, and the proportion of pilot trials that lead to definitive trials will be analyzed. A quantitative analysis of the methodological quality and reporting completeness of the included trials will be implemented by calculating the percentage of items reported in each domain of RoB 2 and CONSORT. The secondary analysis will adopt a regression analysis to identify factors associated with the reporting completeness.

Ethics and dissemination: Ethical approval is not required for this study. This study is planned to be submitted to a peer-reviewed academic journal.

Study registration: Prospective protocol registration is implemented on 12 April 2021 in the Open Science Framework with a unique identification link (https://osf.io/9kmb3/)

For peer review only

Strengths and limitations of this study

- This systematic review protocol will be the first to investigate the status, reporting completeness, and methodological quality of pilot trials in the acupuncture field.
- The reporting completeness of pilot trials will be evaluated using the Consolidated Standards of Reporting Trials (CONSORT) extension for Reporting Randomized Pilot and Feasibility Trials guideline.
- The methodological quality of included trials will be assessed using the Cochrane's updated risk of bias 2.0 (ROB 2) tool.
- There may be a lack of available information to confirm whether a pilot trial has led to a definitive trial in some included studies.
- Findings from this study will be restricted to trials published between 2011 and 2021.

1. Introduction

Although clinical trials serve as the basis for improvement of clinical practice and establishment of evidence-based clinical guidelines, they generally consume considerable human, financial, and material resources. Moreover, it is difficult to make major changes once they are conducted formally[1]. In such situations, if the design of the formal trial has major flaws, it will lead to a waste of resources. A previously published study[2] estimated that 85% of funds for biomedical research are wasted each year due to misdirected or poorly designed studies. Therefore, it is of significance to validate and improve scientific questions, trial designs, and protocol implementations by conducting pilot trials before formal trials[3].

Despite that several definitions of pilot trials have been put forward, most of them share the idea of conducting a study prior to a larger, more comprehensive study[4]. For example, the UK National Institute for Health Research (NIHR) defines a pilot trial explicitly as 'a version of the main study that is run in miniature to test

For peer review only - http://bmjopen.bmj.com/site/about/guidelines.xhtml

whether the components of the main study can all work together', which is a widely accepted definition[4]. Therefore, whether a study can be labeled as a pilot trial mainly depends on the primary objective of the study, which is to evaluate the feasibility and coordination of the components of an ensuing definitive trial.

Given that the design of a pilot trial is generally consistent with the subsequent formal trial, a well-designed pilot trial can not only identify potential problems that arise in a formal trial in advance but also promote the formal trial by investigating the feasibility and coordination of the study design, thereby avoiding potentially disastrous consequences if large-scale trials are conducted rashly[5]. In the guidelines of the UK Medical Research Council (MRC) for designing and evaluating complex interventions, pilot studies are explicitly recommended, particularly for identifying problems that might arise in a subsequent large-scale trial of a complex intervention[6, 7].

Acupuncture belongs to complex interventions, so it is more necessary to conduct pilot trials before a formal acupuncture trial. In recent years, the benefits of pilot trials have been verified in a number of high-quality clinical trials of acupuncture. For example, based on results obtained from a series of pilot trials involving acupuncture for treating osteoarthritis[8-10], Liu et al. not only fully examined the study procedure to determine the feasibility of study designs, but also obtained important pilot data, such as optimal acupuncture treatment frequency, patient compliance rate, and primary outcome measures. Subsequently, their research team was able to improve the study design in the large-scale definitive trial and implemented it successfully, which was published in the high-impact journal *Arthritis & Rheumatology*[11].

Despite notable value and impacts, the role of pilot studies remains underestimated in scientific research. Moreover, previous studies demonstrated that only a very small percentage of pilot trials lead to definitive trials[12]. To the best of our knowledge, there have been no published studies that aimed to investigate the

For peer review only - http://bmjopen.bmj.com/site/about/guidelines.xhtml

status and quality of pilot trials in the acupuncture field. Key questions concerning the publication trend, impact, feasibility, reporting completeness, and methodological quality of acupuncture pilot trials are urgent to be solved. Thus, this systematic review protocol is designed and it aims to: 1) investigate the overall publication trends and the aspects of feasibility evaluated in acupuncture pilot trials; 2) identify the proportion of acupuncture pilot trials that lead to definitive trials; 3) assess the reporting completeness and methodological quality of pilot trials in the acupuncture field.

2. Methods

This systematic review protocol is drafted according to the Preferred Reporting Items for Systematic Reviews and Meta-Analyses for Protocols (PRISMA-P) guidelines.

2.1 Study registration

This protocol has been prospectively registered on 12 April 2021 in the Open Science Framework with a unique identification link (https://osf.io/9kmb3/).

2.2 Eligibility criteria

A previously published tutorial[5] regards that 'pilot' trials belong to the dimension of 'feasibility' trials and a pilot trial is synonymous with a feasibility study that aims to guide the planning of a large-scale investigation. Based on the main goals of this review and references of similar studies[13-15], the boundary between pilot and feasibility trials will not be imposed in our study. We use the term 'pilot' trial to refer to studies that will be reviewed here and the term 'feasibility' if the original authors of the included publications adopted this term rather than 'pilot'. Therefore, published trials will be eligible if studies were defined and reported explicitly as pilot trials within the published paper itself by the original authors. Additionally, as has been done previously in similar studies[13-15], publications labeled as 'feasibility',

For peer review only - http://bmjopen.bmj.com/site/about/guidelines.xhtml

'preliminary', 'exploratory', 'proof-of-concept' and 'vanguard' studies/trials' will also be considered for eligibility because these terms are often used interchangeably with the term 'pilot'[12, 16, 17]. In addition, given that the International Committee of Medical Journal Editors proposed that all trials conducted after July 2005 should be registered in a public registry platform[18, 19] and the latest update of the Consolidated Standards of Reporting Trials (CONSORT) statement was released in 2010[20], only publications of pilot trials published after 2010 will be evaluated for eligibility in our review.

Studies reported as acupuncture pilot or feasibility trials will be eligible for this review if they meet the following criteria: 1) included the evaluation of at least one kind of acupuncture intervention; 2) randomized control trials (RCTs) conducted in humans; 3) trials published from 2011 to 2021 in any language. The term 'acupuncture' in this review is defined as interventions involving penetration of specific points on the skin by needling, regardless of needling and stimulation modalities, such as manual acupuncture, electroacupuncture, auricular acupuncture, and warm needling, or a combination of these modalities. Studies will be excluded if: 1) they are non-RCTs or there are no control groups, such as trials with a pre-post design or an observational study design; 2) no acupuncture intervention was used solely or in combination in the trial.

2.3 Databases and search strategy

To search published studies of acupuncture pilot trials, we will conduct a comprehensive retrieval on 4 English databases (PubMed, Web of Science, EMBASE, Cochrane Library) and 3 Chinese databases (Chinese National Knowledge Infrastructure, Wanfang Database, Chinese Biomedical Literature Database) in January 2022. The time range of published studies will be limited from 2011 and 2021.

Search strategies will consist of two components: interventions (manual

For peer review only - http://bmjopen.bmj.com/site/about/guidelines.xhtml

acupuncture, electroacupuncture, etc) and study type (pilot trial). Search strategies will combine subject headings (e.g. Medical Subject Headings [MeSH] for PubMed) and keywords (acupuncture, eletroacupuncture, pilot, feasibility, preliminary, exploratory, proof-of-concept, vanguard, etc). The PubMed retrieval strategy will be adapted for other databases by replacing MeSH terms with proper subject headings (if applicable) and maintaining the same keywords. The retrieval strategy for PubMed is presented in Table 1.

2.4 Screening and study selection

All retrieval records identified from 7 electronic databases will be exported into Mendeley Reference Manager software (Elsevier Publishing, 2013) for screening. Following the removal of duplicate retrieval literature using Mendeley, two investigators (YJZ and HTH) will scan titles and abstracts of retrieval studies independently to determine studies that claim to be acupuncture pilot or feasibility trials. In the first round of screening, two independent investigators will browse the titles and abstracts of publications to confirm eligibility. In the second round of screening, the full text of potentially qualified studies will be downloaded to determine eligibility based on the inclusion criteria and exclusion criteria. A third senior investigator (JQF) will be invited to solve any disagreements between the two investigators during study selection. Additionally, if the number of all excluded studies is ≤ 500, a description of the specific reasons for each excluded study will be provided in an Excel sheet. If the total number is more than 500, the reference lists of excluded studies will be supplied in an online appendix. The flowchart of study search and screening is presented in Figure 1.

2.5 Data extractions

A pre-designed electronic data extraction form using Microsoft Excel (Microsoft Corporation, 2018) will be developed for this study based on the recommendation

For peer review only - http://bmjopen.bmj.com/site/about/guidelines.xhtml

from the CONSORT group[20]. Two investigators (YJZ and HTH) will extract data of all included trials independently using the data extraction form, which consists of various aspects such as 1) the publication title; 2) the name of the first author and corresponding author; 3) the number of authors; 4) the language of publication; 5) the country that conducts the trial; 6) the publication year; 7) study designs; 8) conditions being treating; 9) types of intervention; 10) types of controls; 11) trial registration platforms (if applicable); 12) the impact factor of the journal (if applicable) in the publication year based on the Journal Citation Reports (https://jcr.incites.thomsonreuters. com); 13) sample sizes; 14) sources of funding; 15) outcome measures; and so on.

With the attempt to improve consistency and accuracy between the investigators, prior to full-scale data extraction, the feasibility of data extraction forms and between-reviewer agreements will be investigated via kappa coefficients by choosing 10 randomly selected studies. If inadequate consistency and accuracy are identified, extra training on data extraction will be conducted. A third senior reviewer (JQF) will be invited to verify the accuracy of all extracted data and settle any discrepancies between the two investigators.

For peer review only

2.6 Assessment of the aspects of feasibility

(1) Rationales for conducting a pilot trial.

We will classify whether the original authors of the pilot study stated the rationales for conducting a pilot trial (1 for yes; 0 for no). If yes, we will further classify the rationales for conducting pilot and feasibility studies based on a widely acknowledged tutorial, which suggests 4 broad classifications to sort out the rationales for performing pilot trials as follows[5].

1) Process: this involves the assessment of the feasibility of the steps that need to take place as part of the future definitive trial, such as estimation of recruitment rates, retention rates, etc.

For peer review only - http://bmjopen.bmj.com/site/about/guidelines.xhtml

2) Resources: concerning the evaluation of budget and time problems that might arise during the future definitive trial.

3) Management: this involves the evaluation of potential human and data optimization problems, such as personnel and data management issues in different research centers.

4) Scientific: this involves the evaluation of treatment safety, determination of treatment dose, and estimation of treatment effect size, such as the acquisition of preliminary data for sample size estimation in the future definitive trial

If there is more than one classification identified for some included studies, all applicable classifications will be coded.

(2) Conclusions of a pilot trial

In general, the conclusions of a pilot or feasibility trial can be grouped under several classifications as follows[15]: 1) the study protocol is not feasible and the future full-scale definitive trial is not recommended to be conducted; 2) the study protocol is feasible to a certain extent but needs to be modified before proceeding to a future full-scale definitive trial; 3) the study protocol does not need to be modified, but the future full-scale definitive trial needs to be performed under close monitoring; 4) the study protocol is completely feasible. We will classify the conclusion of each included pilot trial according to the above-mentioned classifications.

(3) Whether a future definitive trial is conducted

The strategies for identifying potential definitive trials as far as possible in a similar study[13] will be utilized in our systematic review. In detail, with all the included pilot trials determined, a secondary search will be performed in the same databases to identify corresponding definitive trials. In addition, the International Clinical Trials Registry Platform (ICTRP), Clinicaltrials Registry (https://clinicaltrials.gov/), and Chinese Clinical Trial Registry (http://www.chictr.org.cn) will be searched to ascertain whether a definitive trial was conducted (or in progress). The search terms for definitive trials in databases and

For peer review only - http://bmjopen.bmj.com/site/about/guidelines.xhtml

trials registry platforms are based on some unique information of the published pilot studies (e.g. the name of the corresponding authors, the name of one or more co-authors, the major part of the article title). Finally, if no definitive trials are identified based on these retrieval methods, we will try to contact the corresponding authors of the published pilot study by email and query whether a subsequent definitive trial was in progress, conducted, or submitted for publication.

With the obtained results, we will classify whether a definitive trial was conducted following the pilot trial (1 for yes; 0 for no) and calculate the proportion of pilot trials that lead to definitive trials. Meanwhile, we will investigate whether the pilot data were used for sample size calculations in the subsequently published definitive trial (1 for yes; 0 for no).

2.7 Reporting completeness assessment of pilot trials

The reporting completeness of each included study will be evaluated in accordance with the CONSORT extension for reporting randomized pilot and feasibility trials, which consist of a 40-item checklist[21]. The CONSORT checklist mainly consists of multiple items regarding titles, abstracts, objectives, trial designs, methods, results, conclusions, limitations, registrations, and fundings[22]. In our study, each item of the CONSORT checklist will be scored as ‘reported’, ‘inadequately reported’, ‘not reported’ or ‘not applicable’, as has been done previously in a similar study in other research filed[14]. Reporting completeness assessment will be performed by two independent investigators (XYL and JLL). Any disagreement between the two reviewers will be solved through discussion or be arbitrated by a third senior reviewer (YLJ) until a consensus is reached.

2.8 Methodological quality assessment of pilot trials

The methodological quality of the included studies will be assessed by two independent reviewers (XYL and JLL) using the Cochrane collaboration’s updated

For peer review only - http://bmjopen.bmj.com/site/about/guidelines.xhtml

risk of bias 2.0 (RoB 2.0) tool, in which the ROB of each included trial will be assessed based on the following domains: 1) Randomization process; 2) deviations from intended interventions; 3) missing outcome data; 4) measurement of the outcome; and 5) selection of the reported result. Each domain will be rated as ‘low’, ‘high’, or ‘some concerns’. The overall ROB for a single trial will be also classified as ‘low’ (ROB is low for all domains), ‘some concerns’ (some concerns in at least one domain), and ‘high’ (high ROB for at least one domain or some concerns for multiple domains). Disagreements between the two independent reviewers will be judged by the third senior reviewer (JQF).

2.9 Statistical analysis

For the primary analysis, the number of publications per year will be calculated and presented in a tendency chart to analyze overall publication trends. The aspects of feasibility assessed in the included pilot trials will be tabulated. The proportion of pilot trials that lead to definitive trials will be calculated. A quantitative analysis of the reporting completeness and methodological quality of all included acupuncture pilot trials will be conducted. In detail, the frequency that each item is scored as ‘reported’, ‘inadequately reported’, ‘not reported’, and ‘not applicable’ for the CONSORT checklist will be tabulated; the summary score will be calculated as mean ± standard deviation (SD). The frequency that each domain is rated as ‘low’, ‘high’, or ‘some concerns’ for ROB 2.0 will be tabulated.

For the secondary analysis, regression analysis will be applied to determine which study characteristic (e.g. language of publication, the number of authors, trial registration or not, trial funded or not) has associations with the summary score for the CONSORT checklist. A p-value less than 0.05 is considered significant. All analyses will be performed using SPSS 25.0 software (IBM, USA).

For peer review only - http://bmjopen.bmj.com/site/about/guidelines.xhtml

2.10 Patient and public involvement

Patients and the public will not involve in this study.

2.11 Ethics and dissemination

Ethical approval is not required for this study because no personal and private information of individuals will be involved. This study is planned to be submitted to a peer-reviewed academic medical journal.

3. Discussion

The pilot trial is a key component of clinical research to obtain important preliminary data and information for improving the study design and methodological quality of future definitive trials[5]. Based on a brief review of acupuncture studies published in journals with high impact factors in recent years, the majority of them are conducted on the basis of earlier published pilot trials. For example, Liu et al. conducted an acupuncture pilot trial[23] and found that electroacupuncture at Zhongliao (BL33) and Huiyang (BL35) could lead to less urine leakage in women of stress urinary incontinence with an acceptable safety and feasibility profile. This pilot trial[23] provided an adequate basis for the acupoint selection and feasibility for a subsequent multicenter RCT published in *JAMA*[24]. Additionally, Liu et al. performed a series of pilot trials investigating the effect of acupuncture on functional constipation[25-27], which serve as a solid basis for the subsequent definitive trial of acupuncture for chronic severe functional constipation published in *Annals of Internal Medicine*[28]. Such situations indicate that acupuncture researchers are paying increasing attention to the value of pilot trials.

Moreover, acupuncture belongs to complex treatment interventions and it is a key component of complementary and alternative medicine. Due to the unique nature of acupuncture operations, some patients with specific diseases may be unwilling or unable to receive acupuncture treatment, especially in western countries. Thus, before

For peer review only - http://bmjopen.bmj.com/site/about/guidelines.xhtml

the formal trial, preliminary data are very valuable. For example, it is crucial to obtain preliminary data regarding the recruitment rate, dropout rate, and treatment frequency prior to a subsequent definitive trial. The interpretation of these preliminary results will directly determine whether a future full-scale formal trial should be conducted, or whether the current study protocol needs improvement. It can also provide pilot data for sample size calculations in the subsequent definitive trial. In such a scenario, pilot trials play a crucial role in ensuring a feasible and sound methodological approach in the future definitive trial.

To date, however, there has been a great lack of knowledge about the publication trend, reporting completeness, and methodological quality of acupuncture pilot trials. To the best of our knowledge, there have been no previously published similar studies in the acupuncture field. This will be the first systematic review protocol that aims mainly to investigate the status, reporting completeness, and methodological quality of pilot RCTs in the acupuncture field. Therefore, this study is of significance, which will guide acupuncture clinical research for both researchers and clinicians in the future.

It is worthnoting that our study has several strengths. The first strength is to conduct a broad retrieval to identify all eligible acupuncture pilot trials published from 2011 to 2021 in 7 mainstream electronic databases. Subsequently, the status and overall publication trends will be revealed. Secondly, this systematic review will assess the reporting completeness of each included pilot RCT following the 40-item CONSORT extension for reporting randomized pilot and feasibility trials[21], which is a highly acknowledged and recommended tool[29]. Transparent and complete reporting is a key principle of rigorous research, and reporting tools (eg, CONSORT) enable both authors and readers to interpret studies. Specifically, the CONSORT extension for reporting randomized pilot and feasibility trials[21] is developed to provide reporting guidance for any randomized study in which a future definitive RCT, or part of it, is conducted on a smaller scale, regardless of its design (eg, cluster, crossover)

For peer review only - http://bmjopen.bmj.com/site/about/guidelines.xhtml

or the terms used by authors to label the study (eg, pilot, feasibility). A previous systematic review indicated that the application of the CONSORT checklist has close associations with improvement in the proper reporting of RCTs[30]. Thus, findings of our systematic review will provide a better understanding of the reporting completeness and transparency of current studies. On the basis, it will further shed the light to promote the appropriate reporting of acupuncture pilot trials in the future. Third, the methodological quality of each included pilot RCT will be assessed using Cochrane's updated ROB 2.0 tool[31], which is a well-established and reliable method. A previous study[32] has proved its fair reliability and good construct validity and ROB 2.0 is also commonly used in systematic reviews with similar research goals [33-34].

Nevertheless, several limitations of this study should be addressed. First, despite that multiple measures will be adopted to identify subsequent definitive trials on the basis of the included pilot studies, there may be a lack of available information to confirm whether an acupuncture pilot trial has led to a definitive trial in some included studies. Second, given that the CONSORT extension for reporting randomized pilot and feasibility trials is applied to evaluate the reporting completeness and transparency of included studies and the latest update of the CONSORT statement was released in 2010[20], only acupuncture pilot RCTs published from 2011 to 2021 will be included for analysis in our review. Third, it is difficult to clearly distinguish between the small studies and pilot studies in some situations. Some clinical studies are conducted based on previous small studies, but the terms 'pilot' or 'feasibility' probably don't appear in these published small studies. It could affect the comprehensiveness of literature retrieval.

For peer review only - http://bmjopen.bmj.com/site/about/guidelines.xhtml

References

[1] Wang Y, Wang LQ, Yang JW, et al. Application and reflection on pilot study in acupuncture clinical research. Chin Acup Moxib. 2021;41(3):325-329.

[2] Chalmers I, Glasziou P. Avoidable waste in the production and reporting of research evidence. Lancet. 2009;374(9683):86-89.

[3] Moher D, Glasziou P, Chalmers I, et al. Increasing value and reducing waste in biomedical research: who's listening?. Lancet. 2016;387(10027):1573-1586.

[4] Eldridge SM, Lancaster GA, Campbell MJ, et al. Defining Feasibility and Pilot Studies in Preparation for Randomised Controlled Trials: Development of a Conceptual Framework. PLoS One. 2016;11(3):e0150205.

[5] Thabane L, Ma J, Chu R, et al. A tutorial on pilot studies: the what, why and how. BMC Med Res Methodol. 2010;10:1.

[6] Craig P, Dieppe P, Macintyre S, et al. Developing and evaluating complex interventions: the new Medical Research Council guidance. Bmj. 2008;337:a1655.

[7] Lancaster GA, Campbell MJ, Eldridge S, et al. Trials in primary care: statistical issues in the design, conduct and evaluation of complex interventions. Stat Methods Med Res. 2010;19(4):349-377.

[8] Lin LL, Li YT, Tu JF, et al. Effectiveness and feasibility of acupuncture for knee osteoarthritis: a pilot randomized controlled trial. Clin Rehabil. 2018;32(12):1666-1675.

[9] Lin LL, Tu JF, Wang LQ, et al. Acupuncture of different treatment frequencies in knee osteoarthritis: a pilot randomised controlled trial. Pain. 2020;161(11):2532-2538.

[10] Wang TQ, Li YT, Wang LQ, et al. Electroacupuncture versus manual acupuncture for knee osteoarthritis: a randomized controlled pilot trial. Acupunct

For peer review only - http://bmjopen.bmj.com/site/about/guidelines.xhtml

Med. 2020;38(5):291-300.

[11] Tu JF, Yang JW, Shi GX, et al. Efficacy of Intensive Acupuncture Versus Sham Acupuncture in Knee Osteoarthritis: A Randomized Controlled Trial. Arthritis Rheumatol. 2021;73(3):448-458.

[12] Arain M, Campbell MJ, Cooper CL, et al. What is a pilot or feasibility study? A review of current practice and editorial policy. BMC Med Res Methodol. 2010;10:67.

[13] Desai B, Desai V, Shah S, et al. Pilot randomized controlled trials in the orthopaedic surgery literature: a systematic review. BMC Musculoskelet Disord. 2018;19(1):412.

[14] Scola LFC, Moseley AM, Thabane L, et al. A methodological survey on reporting of pilot and feasibility trials for physiotherapy interventions: a study protocol. BMJ Open. 2019;9(5):e020580.

[15] Kaur N, Figueiredo S, Bouchard V, et al. Where have all the pilot studies gone? A follow-up on 30 years of pilot studies in Clinical Rehabilitation. Clin Rehabil. 2017;31(9):1238-1248.

[16] Arnold DM, Burns KE, Adhikari NK, et al. The design and interpretation of pilot trials in clinical research in critical care. Crit Care Med. 2009;37(1 Suppl):S69-74.

[17] Leon AC, Davis LL, Kraemer HC. The role and interpretation of pilot studies in clinical research. J Psychiatr Res. 2011;45(5):626-629.

[18] De Angelis C, Drazen JM, Frizelle FA, et al. Clinical trial registration: a statement from the International Committee of Medical Journal Editors. N Engl J Med. 2004;351(12):1250-1251.

[19] Costa LO, Lin CW, Grossi DB, et al. Clinical trial registration in physical therapy journals: Recommendations from the International Society of Physiotherapy Journal Editors. Phys Ther. 2013;93(1):6-10.

[20] Schulz KF, Altman DG, Moher D. CONSORT 2010 statement: updated

For peer review only - http://bmjopen.bmj.com/site/about/guidelines.xhtml

guidelines for reporting parallel group randomised trials. BMJ. 2010;340:c332.

[21] Eldridge SM, Chan CL, Campbell MJ, et al. CONSORT 2010 statement: extension to randomised pilot and feasibility trials. Pilot Feasibility Stud. 2016;2:64.

[22] Thabane L, Hopewell S, Lancaster GA, et al. Methods and processes for development of a CONSORT extension for reporting pilot randomized controlled trials. Pilot Feasibility Stud. 2016;2:25.

[23] Xu HF, Du RS, Mo Q, et al. A phase 1 clinical study on the efficacy of electroacupuncture for female patients with mild to moderate stress incontinence. China Journal of Traditional Chinese Medicine and Pharmacy. 2014;29(12):3755-3758.

[24] Liu Z, Liu Y, Xu H, et al. Effect of Electroacupuncture on Urinary Leakage Among Women With Stress Urinary Incontinence: A Randomized Clinical Trial. JAMA. 2017;317(24):2493-2501.

[25] Xue QM, Li N, Liu ZS, et al. Efficacy of electroacupuncture in the treatment of functional constipation: A randomized controlled pilot trial. Chin J Integr Med. 2015;21(6):459-463.

[26] Qu JN, Zhang BY, Xu HF, et al. Effi cacy of electro-acupuncture for severe functional constipation: A randomized controlled pilot trial. China Journal of Traditional Chinese Medicine and Pharmacy. 2014;29(5):1424-1428.

[27] Qu JN, Zhang BY, Zhu WZ, et al. Comparison of efficacy on functional constipation treated with electroacupuncture of different acupoint prescriptions: a randomized controlled pilot trial. Chin Acup Moxib. 2014;34(6):521-528.

[28] Liu Z, Yan S, Wu J, et al. Acupuncture for Chronic Severe Functional Constipation: A Randomized Trial. Ann Intern Med. 2016;165(11):761-769.

[29] Thoma A, Coroneos CJ, Eaves FF. You Can't See What You Can't See: Transparency in RCT Reporting, and the Role of the CONSORT Checklist. Aesthet Surg J. 2021;41(6):741-743.

For peer review only

[30] Plint AC, Moher D, Morrison A, et al. Does the CONSORT checklist improve the quality of reports of randomised controlled trials? A systematic review. Med J Aust. 2006;185(5):263-267.

[31] Sterne JAC, Savović J, Page MJ, Elbers RG, Blencowe NS, Boutron I, et al. RoB 2: a revised tool for assessing risk of bias in randomised trials. BMJ. 2019;366:l4898.

[32] Bühn S, Mathes T, Prengel P, et al. The risk of bias in systematic reviews tool showed fair reliability and good construct validity. J Clin Epidemiol. 2017;91:121-128.

[33] Lu T, Lu C, Li H, Xing X, Deng X, Li X, et al. The reporting quality and risk of bias of randomized controlled trials of acupuncture for migraine: Methodological study based on STRICTA and RoB 2.0. Complement Ther Med. 2020;52:102433.

[34] Young A, Reeves BC, Cheng HY, Wasiak J, Muir D, Davies A, et al. Risk of bias and reporting completeness of randomised controlled trials in burn care: protocol for a systematic review. BMJ open. 2019;9(12):e033472.

For peer review only - http://bmjopen.bmj.com/site/about/guidelines.xhtml

Contributors

HTH and JQF were responsible for the conception and design of the protocol. XFH and YLJ provided critical insights into the methodology. YJZ drafted the first version of the manuscript. JLL and XYL revised the manuscript. All authors have critically reviewed and approved the final manuscript.

Funding

The trial is financially supported by the National Key R&D Program of China (NO.2018YFC1704600).

Competing interests

The authors declare that they have no competing interests.

Data availability statement

All data are available upon reasonable request

For peer review only - http://bmjopen.bmj.com/site/about/guidelines.xhtml

Figure legends

Figure 1. The flow diagram of study search and screening

For peer review only

Table 1. Search strategy in PubMed.

No.	Search items
#1	Randomized controlled trial [pt]
#2	Controlled clinical trial [pt]
#3	Randomized OR Randomised [Title/Abstract]
#4	Clinical trials [MeSH]
#5	Randomly [Title/Abstract]
#6	Trial [Title/Abstract]
#7	#1 OR #2 OR #3 OR #4 OR #5 OR #6
#8	Pilot Projects OR Feasibility Studies OR Proof of Concept Study [MeSH]
#9	pilot OR feasibility OR preliminary OR exploratory OR proof-of-concept OR vanguard [Title/Abstract]
#10	#8 OR #9
#11	Acupuncture Therapy [MeSH]
#12	(acupuncture OR electroacupuncture OR electro-acupuncture OR manual acupuncture OR auricular acupuncture OR ear acupuncture OR warm needling OR warming-needle moxibustion) [Title/Abstract]
#13	#11 OR #12
#14	#7 AND #10 AND #13

For peer review only - http://bmjopen.bmj.com/site/about/guidelines.xhtml

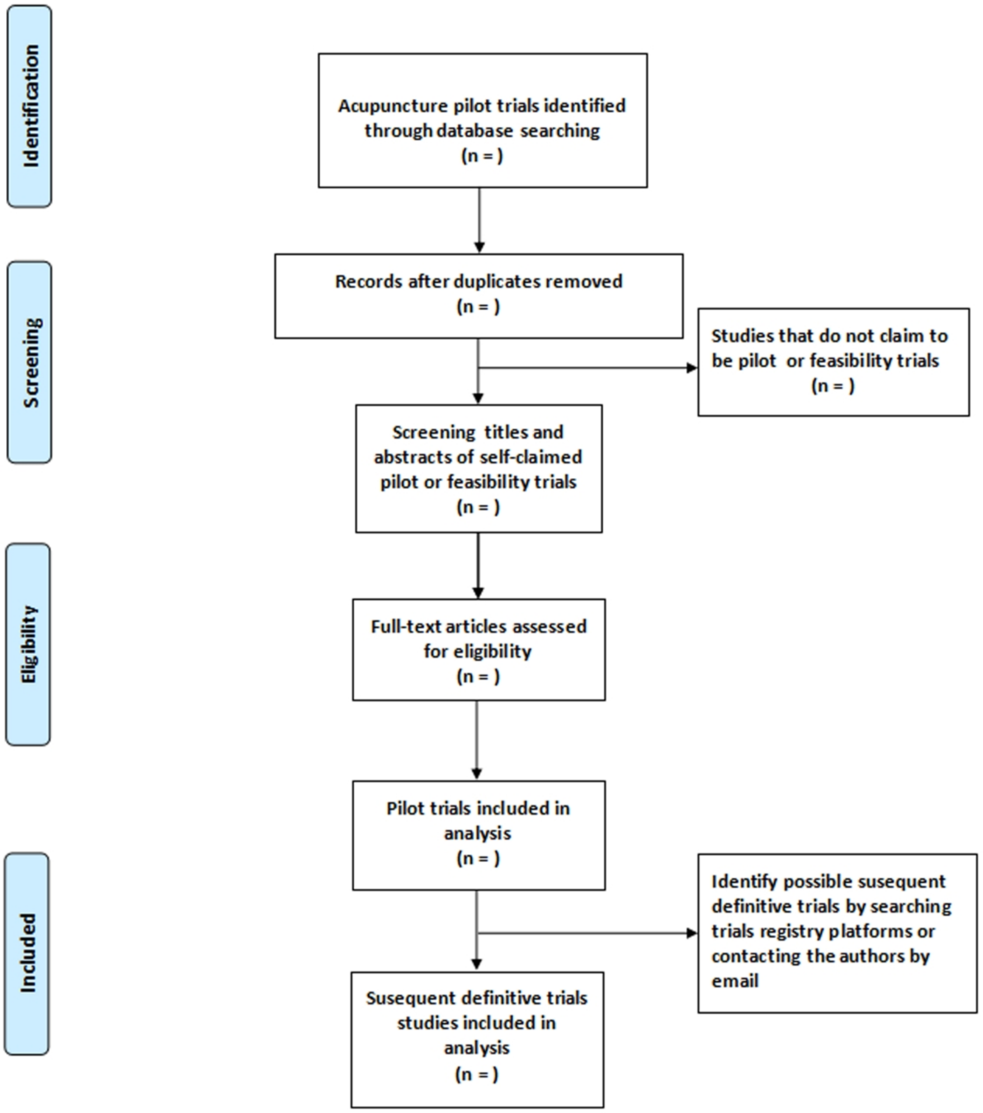

Figure 1. The flow diagram of study search and screening

226x256mm (300 x 300 DPI)

PRISMA-P (Preferred Reporting Items for Systematic review and Meta-Analysis Protocols) 2015 checklist: recommended items to address in a systematic review protocol*

Section and topic	Item No	Checklist item	Reported on Page #
**ADMINISTRATIVE INFORMATION**			
Title:			1
Identification	1a	Identify the report as a protocol of a systematic review	1
Update	1b	If the protocol is for an update of a previous systematic review, identify as such	NA
Registration	2	If registered, provide the name of the registry (such as PROSPERO) and registration number	5
Authors:			1
Contact	3a	Provide name, institutional affiliation, e-mail address of all protocol authors; provide physical mailing address of corresponding author	1
Contributions	3b	Describe contributions of protocol authors and identify the guarantor of the review	19
Amendments	4	If the protocol represents an amendment of a previously completed or published protocol, identify as such and list changes; otherwise, state plan for documenting important protocol amendments	NA
Support:			19
Sources	5a	Indicate sources of financial or other support for the review	19
Sponsor	5b	Provide name for the review funder and/or sponsor	19
Role of sponsor or funder	5c	Describe roles of funder(s), sponsor(s), and/or institution(s), if any, in developing the protocol	19
**INTRODUCTION**			
Rationale	6	Describe the rationale for the review in the context of what is already known	3-4
Objectives	7	Provide an explicit statement of the question(s) the review will address with reference to participants, interventions, comparators, and outcomes (PICO)	5
**METHODS**			
Eligibility criteria	8	Specify the study characteristics (such as PICO, study design, setting, time frame) and report characteristics (such as years considered, language, publication status) to be used as criteria for eligibility for the review	5-6
Information sources	9	Describe all intended information sources (such as electronic databases, contact with study authors, trial registers or other grey literature sources) with planned dates of coverage	6
Search strategy	10	Present draft of search strategy to be used for at least one electronic database, including planned limits, such that it could be repeated	6-7, Table 1

For peer review only

Study records:			
Data management	11a	Describe the mechanism(s) that will be used to manage records and data throughout the review	7-8
Selection process	11b	State the process that will be used for selecting studies (such as two independent reviewers) through each phase of the review (that is, screening, eligibility and inclusion in meta-analysis)	7, Figure 1
Data collection process	11c	Describe planned method of extracting data from reports (such as piloting forms, done independently, in duplicate), any processes for obtaining and confirming data from investigators	7-8
Data items	12	List and define all variables for which data will be sought (such as PICO items, funding sources), any pre-planned data assumptions and simplifications	7-8
Outcomes and prioritization	13	List and define all outcomes for which data will be sought, including prioritization of main and additional outcomes, with rationale	7-8
Risk of bias in individual studies	14	Describe anticipated methods for assessing risk of bias of individual studies, including whether this will be done at the outcome or study level, or both; state how this information will be used in data synthesis	10-11
Data synthesis	15a	Describe criteria under which study data will be quantitatively synthesised	11
	15b	If data are appropriate for quantitative synthesis, describe planned summary measures, methods of handling data and methods of combining data from studies, including any planned exploration of consistency (such as I^2, Kendall's τ)	11
	15c	Describe any proposed additional analyses (such as sensitivity or subgroup analyses, meta-regression)	11
	15d	If quantitative synthesis is not appropriate, describe the type of summary planned	11
Meta-bias(es)	16	Specify any planned assessment of meta-bias(es) (such as publication bias across studies, selective reporting within studies)	NA
Confidence in cumulative evidence	17	Describe how the strength of the body of evidence will be assessed (such as GRADE)	NA

*** It is strongly recommended that this checklist be read in conjunction with the PRISMA-P Explanation and Elaboration (cite when available) for important clarification on the items. Amendments to a review protocol should be tracked and dated. The copyright for PRISMA-P (including checklist) is held by the PRISMA-P Group and is distributed under a Creative Commons Attribution Licence 4.0.**

From: Shamseer L, Moher D, Clarke M, Ghersi D, Liberati A, Petticrew M, Shekelle P, Stewart L, PRISMA-P Group. Preferred reporting items for systematic review and meta-analysis protocols (PRISMA-P) 2015: elaboration and explanation. BMJ. 2015 Jan 2;349(jan02 1):g7647.

For peer review only
